# Chronic intermittent abdominal pain in young woman with intestinal malrotation, Fitz-Hugh-Curtis Syndrome and appendiceal neuroendocrine tumor: a rare case report and literature review

**DOI:** 10.1186/s12905-015-0274-2

**Published:** 2016-01-16

**Authors:** Alessia Cusimano, Ahmed Mohammed Alaaeldien Beniamin Abdelghany, Andrea Donadini

**Affiliations:** Department of Surgery, Clinica luganese SA, Via Moncucco 10, 6900 Lugano, Switzerland

**Keywords:** Chronic intermittent abdominal pain, Intestinal mal-rotation, Fitz-Hugh-Curtis syndrome, Appendicular neuroendocrine tumor

## Abstract

**Background:**

There are a lot of different causes of abdominal pain; in this case, a young woman suffers from three diseases with similar symptoms. Adult intestinal mal-rotation is a rare condition of deviation from the normal 270° counter clockwise rotation of the midgut resulting in, not only mal-position of the small intestine, but also mal-fixation of the mesentery. Fitz-Hugh-Curtis syndrome is a rare complication of pelvic inflammatory disease; it involves liver capsule inflammation associated with genital tract infection, which is usually caused by Neisseria gonorrhoea and Chlamydia trachomatis. Neuroendocrine tumors are enterochromaffin cell neoplasms that arise from cells of the endocrine (hormonal) and nervous systems; the appendicular one is the most common primary malignant lesion of these tumors, it’s incidence is about 0.3 – 0.9 % of appendectomies done. Just for knowledge, this is the first described case of concomitant presence of all these diseases with clinical symptoms attributable to each one.

**Case presentation:**

40-years-old woman suffers from acute abdominal pain, predominantly on the right quadrants, without abdominal distension, no guarding nor rigidity and normal intestinal peristalsis. She has a long history of abdominal intermittent pain, with cramps every 30–40 min, resolving spontaneously. She was diagnosed as intestinal mal-rotation through computed tomography scan which has evidenced a mobilized intra - peritoneal duodenum with cecum/ascending colon predominately lying on the left side and the small intestine almost entirely lying on the right side of abdomen, without evidence of effusion, edema or signs of intestinal ischemia or infarction. Exploratory laparoscopy demonstrated an inflammatory process in the hepatic-renal space, with bloody adhesions above the liver capsule; this is additional to the typical pelvic inflammatory disease signs (Fitz-Hugh-Curtis syndrome). Appendectomy was performed with histological analysis resulting in appendicular neuroendocrine tumor.

**Conclusions:**

Although the patient has an intestinal mal-rotation which could explain the abdominal painful symptoms, it is not possible to exclude other concomitant causes, such as perihepatitis on pelvic inflammatory disease or neuroendocrine tumors. Even if all these diseases are rarely seen in daily clinical practice, they should be considered in the differential diagnosis of chronic intermittent abdominal pain in a young woman.

## Background

Intestinal Mal-rotation (IM) is defined as any deviation from the normal 270° counter clockwise rotation of the midgut during embryological development, resulting not only in the mal-position of the small intestine but also in the mal-fixation of the mesentery [1]. It is rare to be manifested in adulthood, but it’s difficult to approve the true incidence because it’s manifestation is nonspecific and the index of suspicion of intestinal mal-rotation progressively decreases in the older population, so usually it isn’t considered as a differential diagnosis in the initial evaluation [2]. The symptoms usually are intermittent abdominal pain with acute complications such as internal hernia, volvulus or intestinal obstruction.

Fitz-Hugh-Curtis syndrome is a complication of pelvic inflammatory disease (PID); it involves liver capsule inflammation associated with genital tract infection, which is usually caused by Neisseria gonorrhoea and Chlamydia trachomatis. The main symptoms are acute abdominal pain, usually in right upper quadrant, sometimes associated also with signs of salpingitis; chills, nausea, vomiting, hiccupping and malaise are commonly reported. The incidence ranges from 4 to 14 % in female with PID, reaching 27 % in adolescents cases.

Neuroendocrine tumors (NETs) are enterochromaffin /neuroendocrine cell neoplasms with special secretory granules capacity that may determine a carcinoid syndrome, including symptoms as abdominal pain, diarrea/constipation, nausea/vomiting, jaundice or changing in stool colour. NETs most commonly occur in the gastrointestinal tract (67 %) and bronchopulmonary system (25 %). The appendicular one is the most common primary malignant lesion originating in the appendix, found in 0.3 – 0.9 % of appendectomies done [3].

We report an unusual case of 40-years-old female with a long history of intermittent abdominal pain, especially on the upper right quadrant, already studied by numerous medical centres without certain definitive diagnosis; we detected an intestinal mal-rotation (with intra-peritoneal duodenum and cecum/ascending colon predominately lying on the left side, adjacent to sigmoid colon, with total absence of the colon in the right side), also we diagnosed a Fitz-Hugh-Curtis syndrome which is usually caused by Chlamydia trachomatis and an appendicular NETs. This rare case indicates that, even if the patient has an intestinal mal-rotation, which could explain the abdominal symptoms, it is not possible to exclude another concomitant causes, as C. trachomnatis infection, PID and NETs.

## Case presentation

**Fig. 1:**
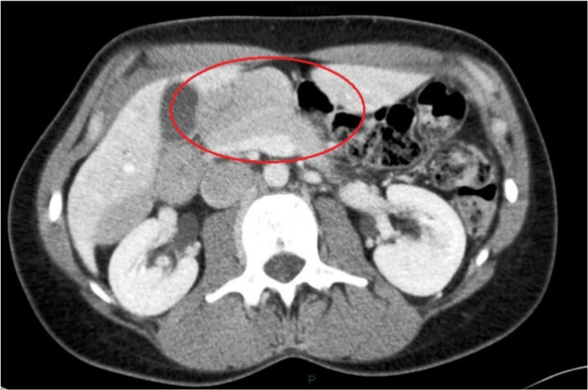
Computed tomography imaging of the intra-peritoneal duodenum (C-shaped portion on the red ring)

**Fig. 2:**
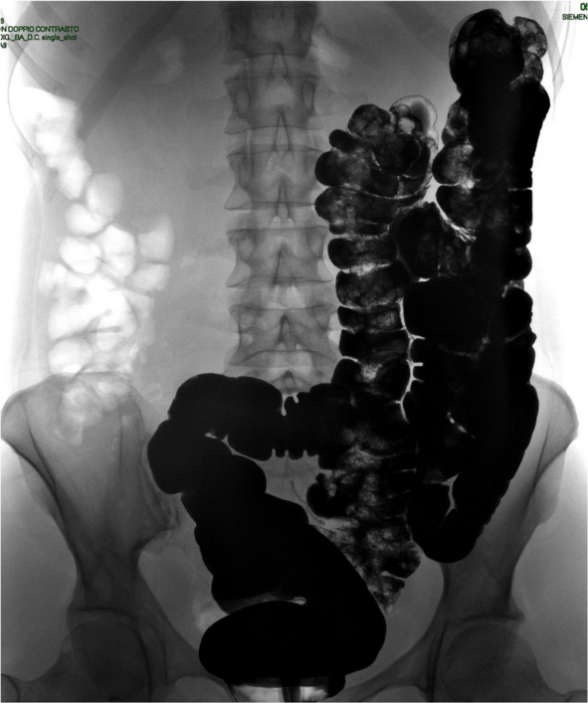
Double-contrast barium enema. The intestinal malrotation with cecum/ascending colon on left side

**Fig. 3:**
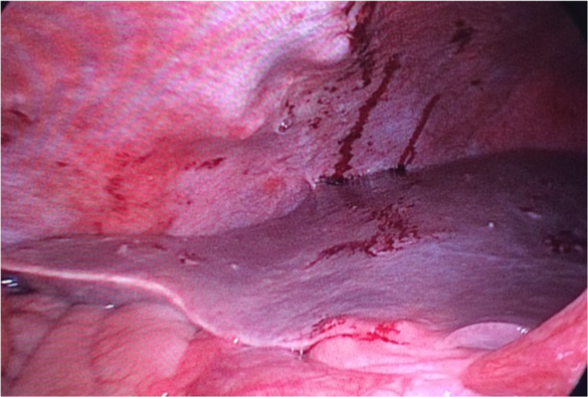
Laparoscopic operative photograph. The bloody perihepatitis associated with adhesions between the liver capsula and abdominal wall

**Fig. 4:**
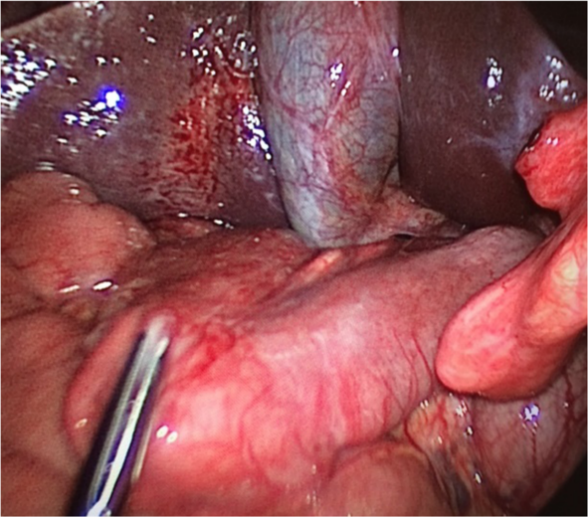
Laparoscopic operative photograph. The perihepatitis and the mobilized and intra-peritoneal duodenum

**Fig. 5:**
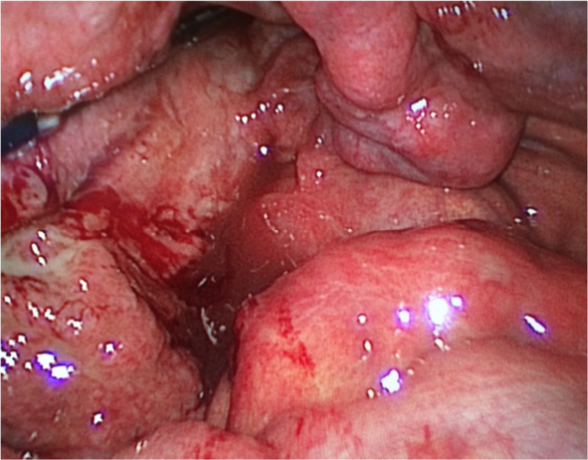
Laparoscopic operative photograph. Macroscopic findings of the PID on the pelvis, with inflammatory disorder of the uterus, right fallopian tube and adjacent pelvic structures

A 40-years-old woman presented to the Emergency Department with acute intermittent abdominal pain with cramps, located in all right quadrants, started from 24 to 48 h. The physical examination results in palpable abdomen without abdominal distension, nor guarding or rigidity and normal intestinal peristalsis; the liver and spleen were not palpable. The clinical evaluation showed absence of fever, nausea, vomiting or modifications of the bowel habit, absence of any abnormal urological symptoms and normal vital signs. All the blood and urine analysis were normal. The history of the patient is free of any abdominal surgical operations. About the sexual history, she was nulliparous, free of dyspareunia, pelvic pain, spotting, itching or other gynaecological symptoms. The patient had in the past some similar episodes of abdominal intermittent pain associated with cramps recurring every 30–40 min, resolving spontaneously, usually two times/year during the past three years; an ultrasound, computed tomography (CT) scan and upper/lower endoscopy have evidenced an intestinal mal-rotation. Now we have repeated the CT scan which has evidenced a mobilized intra-peritoneal duodenum (Fig. 1) in all of its portions associated with the cecum/ascending colon predominately lying on left side and the small bowel almost entirely lying on the right side (Fig. 2), with inverted relationship between superior mesenteric vein (SMV) and artery (SMA) – the vein is lying to the left of the artery. Also evidenced absence of any effusion, edema or signs of intestinal ischemia or infarction. Cause of the persistent pain, recurrence of these episodes, the resistance to standard painkiller drugs and normal infective indicator in blood and urine analysis, we decided to perform an exploratory laparoscopy, which evidenced the colon completely mobilized and located on the left side of the abdomen, the duodenum totally intraperitoneal and well-vascularized, but the presence of intense inflammatory process in the hepatic-renal space with bloody fragile viscero-parietal and viscero-visceral adhesions above the Glisson’s capsule (Figs. 3 and 4), additionally to the typical PID signs, resulting in inflammatory disorder of the uterus, fallopian tubes and adjacent pelvic structures (Fig. 5). Collecting buffers for bacterial culture from the pelvic space and the perihepatic space have been done, then toilette peritoneal lavage. The appendix appeared moderately inflamed, so we performed an appendectomy. After the operation, we immediately began with intravenous antibiotic therapy. The bacterial cultures were positive for C. trachomatis, so we continued with the specific oral antibiotic therapy to be in total 14-days. In the follow-up phase, the patient reported clinical improvement and attenuation of the symptoms. Histological result of the appendix was unexpectedly an appendicular NET (maximum diameter 0,3 cm; pT1a G1); according to the American Joint Commission on Cancer (AJCC) Cancer Staging Manual Seventh Edition, this Stage I just requires follow-up strategy.

## Conclusions

IM is a congenital anomaly, usually diagnosed in the first month of life (nearly 85 % of cases), while it’s rarely found in adulthood (about 0.2–0.5 %); it has been estimated to occur in approximately one in 500 live births, but the true incidence in old age is unknown because the major part of patients are asymptomatic [2]. The common patient manifestation is characterized from acute bowel obstruction, intestinal ischemia, volvulus, chronic intermittent obstruction and non-specific abdominal pain [4]. The intestinal mal-rotation is a deviation from the normal 270° counter clockwise rotation of the midgut. Embryologically, the first stage (Stage I) consists of umbilical cord herniation, lasting from 5^th^ week to the 10^th^ week of embryonic development. The midgut lengthens disproportionately during this period and undergoes rotation around the SMA axis for a total of 270° in the counter clockwise direction. Secondly (Stage II), the midgut loop comes back into the abdomen, approximately from the 10^th^ week to the 11^th^ week; when re-entered into the abdominal cavity, the cephalic midgut completes its 270° counter clockwise rotation while the caudal midgut also completes its rotation, resulting in the duodenum coursing inferior and posterior to the SMA and the caecum located in the right lower quadrant. Stage III lasts from the end of Stage II until just after birth, completing the fixation. The descending and ascending colon mesenteries fuse with the retro peritoneum, the small bowel is fixed by a broad mesentery from the duodenojejunal junction in the left upper quadrant to the ileocecal valve in the right lower abdomen. One of the most frequent forms is “non-rotation”, where the first and second parts of the duodenum are situated normally but the third and fourth parts descending vertically downward along the right side of the superior mesenteric artery, the small bowel on the right and the colon doubled on itself to the left of midline; in “reversed rotation” there is the caecum and colon positioned posterior to the superior mesenteric vessels, the duodenum subsequently crosses anterior to it; finally, “mal-rotation” is a spectrum of abnormalities, the most frequent of which are duodenojejunal flexure located inferiorly in the right of the midline and caecum in a sub hepatic or central position [5]. The anomalies can be classified based on the stage of rotation during which they occur: Stage I anomalies are caused by failure of the gut to return to the abdomen, such as omphaloceles, Stage II abnormalities are “non-rotation”, “mal-rotation” and “reverse rotation”, Stage III abnormalities are unattached duodenum, mobile caecum and unattached small bowel mesentery [6]. If the rotation is 3 incomplete, the caecum remains in the epigastrium and the bands of fixing from the duodenum to the retro peritoneum and the caecum continue to form; they are called Ladd’s bands, starting from the lateral abdominal wall, extending from the caecum and crossing the duodenum, being frequently responsible of intestinal obstruction [2]. The diagnosis is difficult and usually is radiological; conventional radiography is neither sensitive nor specific [2]. Upper gastrointestinal contrast series are the gold standard for paediatrics, while a contrast enhanced CT scan is the most used in adults; CT scan allows to see the inversion of the SMA and SMV, showing usually the vein situated to the left of the artery and/or a vertical relationship. We can obtain a similar result using Doppler sonography, but the CT scan gives us important information like intestinal obstruction, congestion of the mesenteric vasculature, kind of mal-position of the intestine, volvulus, ischemia/necrosis and so on [7]. Clinically, the great part of patients remains asymptomatic; however some of them could present chronic symptoms of intermittent bowel obstruction as in our case. Surgical intervention comes in the form of the Ladd’s procedure: counter clockwise detorsion of the volvulus or other if present, cutting the Ladd’s band, widening of the narrow root of the small bowel mesentery by mobilizing the duodenum and sectioning of the adhesions around SMA [7]. Pelvic inflammatory disease is an infectious inflammatory disorder involving the uterus, fallopian tubes and other pelvic structure, depending on ascending infection from the endocervix; C. trachomatis and N. gonorrhoea are the bacterial agents in approximately 40 % of cases, followed by Mycoplasma genitalium, Trichomonas vaginalis, Herpes simplex type 2; bacterial vaginosis-associated microorganisms and anaerobic organisms have also been isolated, although their role in the complications of pelvic infection remains unclear [8]. Fitz-Hugh-Curtis syndrome is a perihepatitis associated with PID, often difficult to be recognized; it is characterized by inflammation of liver capsule without involvement of hepatic parenchyma. At first, in 1920 Stajano described adhesions between the liver capsule and abdominal wall in a patient with gonococcal infection [9]; but in 1930s Thomas Fitz-Hugh and Arthur Curtis described the syndrome, clarifying the connection between the acute abdominal pain (located on the right upper quadrant) and the pelvic phlogosis with “violinstring” adhesions on evident prior salpingitis [10, 11]. By relying on the consideration that similar adhesions are not present in other peritonitis, Curtis hypothesized a correlation with gynaecological infection by Neisseria gonorrhoea [12]. In 1978, Muller-Schoop et al [13] demonstrated positive cultures of C. trachomatis in woman with perihepatitis; after this and thanks to many other papers, C. trachomatis was recognized as implicated on Fitz-Hugh-Curtis syndrome. Still today, the commonly isolated pathogens are N. gonorrhoeae and C. trachomatis, even if facultative gram-negative rods and anaerobes have been detected [12]. According to the last reviews, a diagnosis of C. trachomatis increased the risk of PID during their reproductive lifetime by 50 %, and each repeated infection increased this risk by a further 20 % [14]. The incidence of this syndrome depends on the criteria used, because it happens that asymptomatic patients have the laparoscopic exploratory indication signs such as symptomatic PID has no evidences on the surgery; usually, it has been attested around 13 % of PID cases, more higher in adolescents (reaching 27 %) [12]. The etiopathogenesis is uncertain; traditionally it’s considered a direct bacterial infection of the liver capsule, but the rare finding of the bacterium in the perihepatic surface or in the peritoneal ascites, the reported syndrome in men, the diffuse abdominal phlogosis, suggest alternative causes (hematogenous spread, lymphatic spread, immune response); none of these alternative aetiologies has clear evidences to support each theory [12]. Diagnosis is difficult based on the atypical abdominal pain (often mistaken for cholecystitis, appendicitis, hepatitis, pleurisy, pyelonephritis and so on), the presence/absence of acute/subacute PID signs and symptoms, aspecific physical signs; radiographic studies give information more to rule out other causes than to confirm the diagnosis; laboratory test are commonly negative or only slightly elevated (electrolytes and liver function test, white blood cell count, urinary levels) but may be useful to confirm the bacteria infection (using vaginal, urine or cervical sample, such as serologic test) [12]. The most frequent symptoms are abdominal pain especially on right upper quadrant (100 %), fever (14.6 %), urinary symptoms (2.4 %), followed by nonspecific symptoms such as chills, sweating, nausea, vomiting, hiccupping, malaise; right upper quadrant abdominal pain is the main symptom, often more severe in response to deep breathing, developing as a result of congestion of hepatic capsules, fibrous exudates and viscero-parietal and viscero-visceral adhesions [15]. The therapy is based on antibiotic regimes. For PID of mild or moderate severity, parenteral and oral therapies appear to have similar clinical efficacy; parenteral therapy can be discontinued 24 h after clinical improvement, but oral therapy should continue to complete 14 days of therapy. Patients who do not respond to oral therapy within 72 h should be re-evaluated to confirm the diagnosis and should be administered parenteral therapy an outpatient or inpatient basis. There are insufficient data to recommend routine use of nonsteroidal anti-inflammatory drugs (NSAIDs) in addiction to antibiotic therapy, in management of PID to reduce inflammatory complications and long-term sequelae; the use of NSAIDs was could reduce tubal obstruction, residual adhesions, pain and overall symptoms but these studies had limited power and were of low quality [8]. In our case, there was urinary positive Chlamydia PCR but negative serology. None sign of ascetic fluid in the hepatic-renal space and pelvis. Laparoscopy provides images of pelvic inflammation and simply bloody peri-hepatic adhesions. The bacterial culture showed positive Chlamydia PCR on peritoneal liquid. Therefore we started antibiotic therapy (Ceftriaxone 2gr daily plus Vibramycin 200 mg daily for the first week, followed by Metronidazole 1000 mg daily plus Vibramycin 200 mg daily for the remaining 7 days).

In our case, in the context of a generalized pelvic inflammation, the appendix appeared moderately inflamed, so we performed a laparoscopic appendectomy; the histological report was of appendicular NET (pT1a G1). The neuroendocrine tumors are so called for the enterochromaffin/neuroendocrine cell origin with neurosecretory capacity, producing and secreting different hormones; many of these products may rise to hormonal syndromes, especially the insulinomas, glucagonomas, gastrinomas and serotoninomas. The appendicular NETs are the most common appendicular tumor, mainly frequent in women (male-to-female ratio 1:2) of 40–50 years old [16]. Appendix is the third commonest site of the gastrointestinal tract (24 %) after small intestine (41.8 %) and rectum (27.4 %); they usually arise in the appendicular tip (70 %), morphologically similar to their small intestinal and rectal counterparts [17]. They may be asymptomatic by themselves and are usually incidentally found during appendectomy for presumed appendicitis, with the majority of cases < 1 cm in diameter (90 %); usually unspecific abdominal pain in the lower right abdomen leads to appendectomy, finding local inflammation and broadening of the appendix which may mimic the same macroscopic pattern of other appendicopathy, so therapeutic decision-making would not be altered [18]. In terms of metastatic potential, they are more benign neoplasm compared to other NETs of the gastrointestinal tract, with much more favourable prognosis. As for all neuroendocrine tumours, risk of metastases is directly related with tumour’s size; it is rare if < 1 cm, 0–1 % between 1 and 2 cm, >20 % in largest dimension. This finding gives the rationale for which patients with appendicular NET ≥2 cm in diameter may benefit from an oncological right hemicolectomy, while in the smaller ones (<1 cm) the therapy is usually appendectomy; decision for NETs 1–2 cm in size is difficult and needs histological criteria and meticulous risk evaluation (benefit/risk ratio) [18]. In these cases, many pathological criteria should be considered, such as serosal or lymph vascular invasion, tumor margins, Ki67 index >2 % on immunohistochemistry and mitotic activity (more than 2 cells per mm2) [19]. As told, the prognosis is better than in other site, with distant metastasis in about 1 % of cases, always in NET >2 cm, and a 5-years survival rate greater than 95 % [17]. At first, the World Health Organization (WHO) classification provides a system for all NET, determining prognosis and treatment, including three main groups subdivided by organ of tumor origin: 1) well differentiated neuroendocrine tumors (benign behavior or uncertain malignant potential-“carcinoids”); 2) well differentiated neuroendocrine carcinomas (low-grade malignancy-“malignant carcinoids”); and 3) poorly differentiated carcinomas (high-grade malignancy) [19]. The appendicular NET are staged by both the AJCC Cancer Staging Manual, 7th edition and the International Union against Cancer (UICC) TNM classification 7th edition [20, 21]; the WHO has harmonized them across gastrointestinal anatomic sites, a system largely in accordance with both the TNM and AJCC [22]. They can also be graded by assessing the mitotic activity or Ki-67 immunolabeling index; Grade 1 tumours (carcinoids) display <2 mitoses/10 high power fields or <2%Ki-67 index and Grade 2 tumours (“atypical carcinoids”) have mitotic counts of 2–20/10 high power fields or 3–20 % Ki-67 index [17]. Intestinal Malrotation is rarely diagnosed in adulthood. Appendicular NET are hardly diagnosed. Fitz-Hugh–Curtis syndrome poses a diagnostic rebus, mimicing many known diseases. Appendicular diseases are not commonly associated to a Fitz-Hugh-Curtis syndrome. In literature, there is just one case report about the concomitant presence of the syndrome and appendicitis, and it has been treated with antibiotics [23]; similarly, a recent case report plus review analysed the co-existence of appendicular NET with endometriosis, considering it so far unique in the literature [24]. No case of appendicular NET and Fitz-Hugh-Curtis Syndrome or PID has been described before. What adds to the exceptionality of our case is that, although the patient has a rare IM that could explain the clinical history, she has other two uncommon diseases, which have the same abdominal signs. Just for knowledge, this is the first reported case of a similar mix of diseases, difficult to be diagnosed due to the rarity of the cases and the non-specificity of the symptoms. Even if all these diseases are rarely seen in daily clinical practice, they should be considered in the differential diagnosis of chronic intermittent abdominal pain in woman. It could represent a clinical *memento*; often the diagnosis is not simple.

## Consent

Written informed consent was obtained from the patient for publication of this Case report and any accompanying images. A copy of the written consent is available for review by the Editor of this journal.

## Abbreviations

AJCC: American Joint Commission on Cancer
CT: computed tomography
IM: intestinal mal-rotation
NET: neuroendocrine tumor
NSAID: nonsteroidal anti-inflammatory drug
PID: pelvic inflammatory disease
SMA: superior mesenteric artery
SMV: superior mesenteric vein
UICC: International Union against Cancer
WHO: World Health Organization
